# Care Setting Trajectories in Last Three Years of Life for People with Dementia

**DOI:** 10.1002/alz.090916

**Published:** 2025-01-09

**Authors:** Olga F Jarrín Montaner, Weiyi Xia, Anum Zafar, Hyosin Kim, Maria J. Lopez, Haiqun Lin

**Affiliations:** ^1^ Rutgers, The State University of New Jersey, New Brunswick, NJ USA; ^2^ Rutgers, The State University of New Jersey, Piscataway, NJ USA; ^3^ Oregon State University, Corvallis, OR USA; ^4^ Rutgers, The State University of New Jersey, Newark, NJ USA

## Abstract

**Background:**

Most older adults prefer aging in place; however, patients with dementia and advanced illness often need institutional care, even if only for a brief period of time. In the context of the aging US population and the increasing number of individuals living with dementia, understanding place of care trajectory patterns is important for patient‐centered care planning and health policy decisions. The purpose of this study was to characterize place of care trajectories during the last three years of life among Medicare beneficiaries diagnosed with dementia.

**Method:**

The analytic cohort included a 20% random sample (n = 186,723) of Medicare beneficiaries who died in 2019 with a diagnosis of dementia, aged fifty or older, and continuously enrolled in Medicare during their last five years of life. Group‐based trajectory modeling was used to identify dual trajectories of inpatient/institutional care, and skilled home healthcare/home hospice.

**Result:**

Ten distinct trajectory classes and their associated sociodemographic and clinical characteristics were identified. The first two classes were characterized by brief use of both skilled home care and institutional care concentrated in the last six‐months of life (40%, 11%); the next three classes were characterized by long‐term use of skilled home healthcare/hospice and low use of inpatient/institutional care (15%, 4%); the next three classes were characterized by long‐term use of inpatient/institutional care and low use of skilled home healthcare/hospice (2%, 2%, 5%), the final three classes were characterized by intermittent use of both institutional and skilled home care during the last two or three years of life (9%, 8%, 3%). We investigate the association between care trajectories and socio‐demographic and clinical factors. Our findings may help to inform dementia care planning and related health and aging policy.

**Conclusion:**

This study identified distinct patterns of care setting trajectories during the last three years of life. While half of older adults with dementia spent their final years at home with minimal use of skilled home care or institutional care until the last six months of life, half had significant skilled care needs over two or more years.

